# Unveiling retrotransposon-derived DNA zip code for myeloma cell internalization

**DOI:** 10.18632/oncoscience.606

**Published:** 2024-07-13

**Authors:** Pavan Kumar Puvvula, Anthony Johnson, Leon Bernal-Mizrachi

**Affiliations:** ^1^Kodikaz Therapeutic Solutions, Inc, New York, NY 10014, USA; ^2^Department of Hematology and Medical Oncology, Winship Cancer Institute of Emory University, Atlanta, GA 30322, USA

**Keywords:** myeloma, non-viral gene therapy, retrotransposons, Rab5a, Clathrin, Syntaxin-6, DNA internalization, Zip-code, cancer

## Background and significance of gene transfer (GT)

The intricate interplay of extracellular genetic material and the tumor genetic landscape poses a significant challenge in understanding cancer evolution, tumor genetic heterogeneity and treatment response. Earlier studies have illuminated the role of circulating tumor DNA (ctDNA) in mediating the gene expression among cancer cells [1–6] shedding light on a previously under-explored avenue of genetic exchange in human malignancies. In this perspective, we delve into the findings of several studies that elucidate the mechanisms underlying ctDNA-driven GT and its potential implications for cancer biology and therapy. GT is a fascinating evolutionary phenomenon observed in lower species and humans, albeit with differing impacts and mechanisms [7]. In lower species, such as bacteria and archaea, Horizontal gene transfer (HGT) plays a pivotal role in their adaptation and survival by facilitating the exchange of genetic material, often through processes like conjugation, transformation, and transduction [8–11]. This process allows them to acquire beneficial traits from their environment or other organisms, such as antibiotic resistance, metabolic capabilities and generally to become fitter organisms.

Horizontal gene transfer in humans can result from various factors, such as viral infections, which can introduce foreign genetic material into our cells [12]. Though less common, these events can have significant implications, contributing to genetic diversity while also leading to the acquisition of new traits or diseases [13, 14]. Overall, HGT underscores the interconnectedness of life forms and highlights the dynamic nature of genetic evolution across diverse species, from the simplest microorganisms to complex organisms like humans. While the prevalence of HGT in humans is still debated, emerging evidence suggests its potential significance in diseases such as metabolic, immunological neurodegenerative diseases and cancer [4, 15].

Earlier studies on HGT in humans were met with skepticism due to the complexity of multicellular organisms› genetic regulation. However, seminal studies provided evidence supporting this hypothesis, including the discovery of endogenous retroviruses (ERVs) integrated into the human genome, indicating ancient viral DNA incorporation [16, 17]. In recent years, studies focusing on blood cancers, such as leukemia and lymphoma, have shed light on the potential role of GT in disease pathogenesis [18]. One striking example is the transfer of genetic material between cancer cells and non-cancerous cells, a phenomenon termed “cancer cell education” or “cancer cell cannibalism [19]”. Several studies demonstrated that leukemia and colon cancer cells could engulf and incorporate genetic material from surrounding cells, increasing tumor heterogeneity and therapeutic resistance [3, 4, 6, 20]. Furthermore, genomic analyses of cancer patients have revealed HGT from pathogens, such as bacteria and viruses, in cancer cells [21]. For instance, a study by Pleasance et al. (2010) identified bacterial DNA integration events in the genomes of leukemia patients, suggesting a potential role of pathogenic DNAs in oncogenesis [22]. Similarly, research by Alvarez et al. uncovered viral sequences integrated into the genomes of hepatocellular carcinoma, implicating viral-mediated HGT in cancer development [23]. The discovery of HGT in human diseases, particularly in the context of cancer [4], has profound implications for our understanding of disease biology and treatment strategies. Harnessing this knowledge could lead to the development of novel therapeutic approaches targeting HGT mechanisms to inhibit disease progression and overcome treatment resistance. However, many questions remain unanswered, warranting further investigation. These include elucidating the extent of HGT in humans, underlying mechanisms, identifying specific causes and conditions and consequences of HGT in cancer. HGT holds immense potential implications for cancer biology and treatment. Here are some possible implications:

1. **Targeted gene delivery:** DNA driven horizontal transfer could offer a novel approach for delivering therapeutic genes directly to cancer cells. By harnessing mechanisms like those observed in natural HGT, such as viral vectors, therapeutic genes could be transferred horizontally into cancer cells, enabling more efficient delivery.
2. **Overcoming genetic heterogeneity:** Cancer is characterized by genetic heterogeneity, where tumor cells within the same tumor exhibit diverse genetic profiles. This heterogeneity leads to difficulties in treatment with targeted therapies not affecting sufficient population of tumor cells to cure the disease. Horizontal gene therapy has the potential to address this heterogeneity by introducing therapeutic genes into large population of cancer cells, regardless of their genetic makeup. This approach could improve treatment efficacy by changing the tumor phenotype to make them susceptible to a drug by expressing a drug target, thereby reducing the likelihood of treatment resistance.
3. **Induction of synthetic lethality:** Horizontal gene therapy could exploit synthetic lethality, a concept where the simultaneous disruption of two genes leads to cell death, to kill cancer cells while selectively sparing normal cells. By horizontally transferring synthetic lethal gene pairs into cancer cells, it may be possible to induce cell death, specifically in cancer cells harboring specific genetic alterations, offering a highly targeted therapeutic approach.
4. **Minimization of off-target effects:** Compared to traditional gene therapy approaches that rely on direct gene insertion, horizontal gene therapy may offer advantages in minimizing off-target effects and unintended genetic modifications. By leveraging natural genetic transfer mechanisms therapeutic genes can be delivered more selectively to cancer cells, reducing the risk of adverse effects on normal tissues.

In GT, the exchange of genetic material between cells occurs through various mechanisms, including direct cell-to-cell contact or extracellular vesicle-mediated transfer [24–30]. We discovered Zip-code DNA sequences through studies demonstrating that ctDNA can transfer genes between cancer cells. We showed that ctDNA naturally has an affinity for recipient cells that are phenotypically and genotypically similar to the donor cells. By identifying the DNA sequences at ctDNA insertion sites and demonstrating that synthetically generated transposon sequences can deliver payloads to target cells, therefore, we proved that these zip code sequences play a pivotal role in HGT communication by mediating targeted interactions between donor and recipient cells, facilitating the transfer of genetic material or signaling molecules [31].

## Triggers of communication between cells in GT

Several factors can trigger communication between cells in GT, including environmental cues, cellular stress, and intercellular signaling molecules [32]. Environmental cues, such as nutrient availability or hypoxia, can modulate the expression or secretion of DNA sequences, influencing their availability for cellular interactions [33]. Cellular stressors, such as DNA damage or inflammation can induce the release of extracellular DNA or RNA, serving as signaling molecules for neighboring cells [34]. Intercellular signaling molecules, including cytokines, growth factors, or microRNAs, can also trigger communication between donor and recipient cells, facilitating GT events [34–37]. The presence of circulating tumor DNA (ctDNA) in the bloodstream can be influenced by various factors [38–43], as mentioned below.

1. **Tumor type and stage:** Different types and stages of cancer can vary in the shedding of ctDNA into circulation. Advanced-stage cancers with higher tumor burdens tend to release more ctDNA compared to early-stage cancers [44–46].
2. **Tumor heterogeneity:** Tumors consist of a diverse population of cancer cells with varying genetic mutations. The shedding of ctDNA into the bloodstream can be influenced by the genetic makeup and heterogeneity of the tumor [47].
3. **Tumor vascularity:** Tumor blood supply and vascularization can affect the release of ctDNA. Highly vascularized tumors with extensive angiogenesis may release more ctDNA due to increased vascular permeability [48].
4. **Treatment status:** Cancer treatments such as surgery, chemotherapy, radiation therapy, targeted therapy, and immunotherapy can impact ctDNA levels. Some treatments may induce tumor cell death and increase ctDNA release, while others may decrease ctDNA levels by reducing tumor burden [49].
5. **Tumor microenvironment:** Factors within the tumor microenvironment, such as hypoxia, inflammation, and immune responses, can affect ctDNA release. Inflammatory processes and immune cell activity may contribute to the release of ctDNA from cancer cells [50].
6. **Patient-specific factors:** Patient characteristics, such as age, overall health, and genetic predisposition, can influence ctDNA levels. For example, patients with compromised immune systems may have altered ctDNA dynamics [51].
7. **Metastatic spread:** The presence of metastases and distant tumor sites can increase ctDNA levels due to DNA shedding from multiple tumor locations [52].

Once released, cell free DNA can be transferred to other cells via different mechanisms including ones independent of a membrane vehicle (soluble fraction) or that are incorporated in extracellular vesicles. Interestingly, only the soluble fraction can affect the host cells phenotype [53].

## Kodikaz therapeutic solutions (KTS)

KTS has explored the mechanisms of GT and presents compelling evidence for the pivotal role of ctDNA as a vehicle for transferring genetic material between cancer cells. Through meticulous experimentation, earlier studies have demonstrated that ctDNA exhibits a remarkable capacity for gene transmission, with a preference for cells resembling its tissue of origin. This discovery challenges conventional views of genetic transfer in human cancers and underscores the significance of ctDNA not only as a means of tumor identification but as a contributor to intra-tumoral heterogeneity and evolution. Detailed molecular characterization revealed that DNA sequences of retro transposons act as mediators of ctDNA GT. Genomic analysis reveals the presence of retrotransposon DNA sequences at ctDNA insertion sites, implicating these mobile elements in facilitating genetic exchange among cancer cells [31]. One of the most intriguing findings is the tissue-specific tropism exhibited by elements of ctDNA, wherein it selectively targets cells resembling its tissue of origin. This phenomenon not only elucidates the mechanisms underlying previous observations of genetic material transfer in cancer models but also holds profound therapeutic implications. The ability to exploit ctDNAs tissue-specific tropism for targeted delivery of therapeutic payloads represents a promising avenue for precision cancer therapy, potentially overcoming challenges associated with systemic drug administration. The research findings of Kodikaz offer significant insights into the mechanisms of cell-free circulating tumor DNA (ctDNA) and its potential implications in cancer biology and other diseases.

## Types and diversity of DNA-based zip code sequences

DNA-based zip code sequences represent diverse genetic elements that facilitate targeted cellular interactions and communication. Understanding the types and diversity of DNA-based zip code sequences, their association with cell receptors and circulatory proteins, and their role in horizontal gene (HG) communication is essential for unraveling the intricacies of intercellular communication and its implications in health and disease. DNA-based zip code sequences encompass genetic motifs, including specific DNA sequences, and nucleic acid ligands, mediating selective interactions with cell receptors and circulatory proteins. These sequences can be naturally occurring or engineered for specific targeting purposes. The diversity of DNA-based zip code sequences offers versatility in target selection and therapeutic design, allowing for tailored approaches in various biomedical applications, including targeted drug delivery, gene therapy, and tissue engineering.

**Figure 1:**
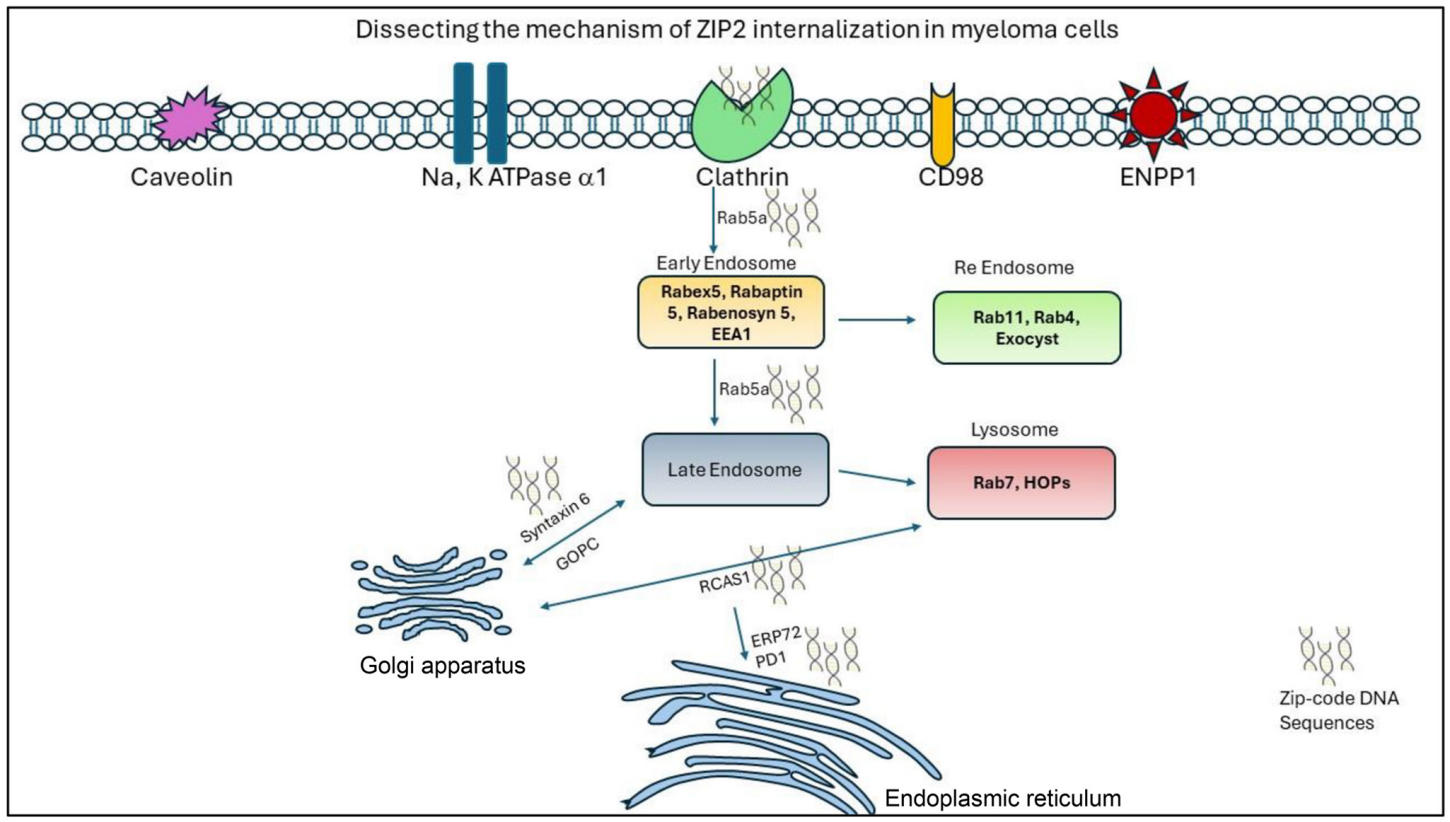
Schematic representation of zip-code DNA internalization in myeloma cells.

Our recently published research [54] sheds light on a novel mechanism by which myeloma cells internalize retrotransposon-derived DNA sequences, offering new insights into the pathogenesis and potential therapeutic targets for multiple myeloma. The study elucidates a previously unrecognized role of retrotransposon-derived DNA elements, in facilitating the internalization of myeloma cells. We have demonstrated that retrotransposon derived from the Alu family act as “zip codes,” guiding the uptake of extracellular DNA by myeloma cells through Clathrin-Rab5a-mediated endocytosis, a cellular process typically associated with nutrient uptake and signaling molecule internalization. By exploiting endogenous cellular machinery for DNA uptake, myeloma cells may acquire genetic material from the microenvironment, potentially influencing tumor progression, drug resistance, and immune evasion. Moreover, identifying Clathrin-Rab5a-mediated endocytosis (Figure 1) as the mechanism underlying retrotransposon-derived DNA internalization provides [54] a rationale for exploring targeted interventions to disrupt this process as a therapeutic strategy for multiple myeloma. Small molecule inhibitors or monoclonal antibodies targeting critical components of the endocytic machinery could impede the uptake of exogenous DNA and attenuate myeloma cell proliferation and survival. Furthermore, the study underscores the importance of understanding the interplay between cancer cells and their microenvironment in shaping tumor behavior and therapeutic responses. Identifying retrotransposon-derived DNA as a potential mediator of cell-cell communication within the tumor microenvironment opens new avenues for investigating the role of extracellular DNA in tumor-stroma interactions, immune modulation, and disease progression.

Capturing foreign DNA involves sequestering or trapping extracellular genetic material, preventing its uptake or integration into recipient cells. Synthetic molecules can be designed to capture foreign DNA and prevent its transfer, thereby limiting cancer cells’ acquisition of oncogenic mutations or drug-resistance genes. Additional evidences demonstrated that nanoparticles functionalized with DNA-binding peptides or aptamers have been explored for their ability to capture circulating tumor DNA (ctDNA) or exosomal DNA released by cancer cells [55, 56]. By sequestering ctDNA or exosomal DNA, these nanoparticles can reduce tumor dissemination, metastasis, and resistance development. Blocking and capturing DNAs prevent the oncogenic transformation, decrease the tumor heterogeneity, and combat the drug resistance [43]. Continued research into mechanisms and therapeutic applications holds the potential to advance cancer treatment and improve patient outcomes.

## Future directions

One of the main efforts should focus on elucidating the precise mechanisms underlying zip code DNA interaction with specific receptors or membrane proteins, and internalization mechanisms by cancer cells. This must be a very dynamic and complicated symphony of interactions but with advances in detection sensitivities of today’s advanced ‘omics and mass spec technologies we are more poised than ever to make these discoveries. By deciphering the molecular interactions between zip code DNAs and cellular receptors or uptake machinery, researchers can engineer targeting motifs for efficient and selective delivery of therapeutic genes to tumor cells while minimizing off-target effects. In addition, incorporating zip code DNAs into non-viral gene delivery vehicles such as lipid nano emulsions, peptide-based vehicles, polymer-based vectors, etc., [57] holds great potential for enhancing their targeting efficiency, stability, and cellular uptake. In this regard, future studies should explore innovative approaches, such as peptide or RNA-based carriers functionalized with zip code motifs, to improve the delivery of therapeutic genes to tumor sites. Developing innovative delivery systems that respond to tumor-specific cues, such as pH or enzyme levels, could further enhance the efficacy of zip code-based gene therapies. Significant challenges in gene therapy involve specific cell targeting, efficient gene transfer, including cellular uptake, endosomal escape, and delivery to specific compartments inside the cell Future research should focus on developing strategies to optimize these steps using zip code DNAs to target moieties. By exploiting endogenous cellular processes, such as Clathrin-mediated endocytosis or nuclear localization signals, we can enhance the intracellular delivery and expression of therapeutic genes in cancer cells. Zip code-based gene therapy strategies can be combined with other therapeutic modalities, such as chemotherapy, radiotherapy, immunotherapy, or targeted therapy, to achieve synergistic effects in cancer treatment. Future research should explore the potential of zip code DNAs to enhance the delivery of cytotoxic agents, radiotherapy, or immunomodulatory molecules to tumor cells, thereby improving therapeutic outcomes. Moreover, the modulation of immune responses using zip code-based gene therapies, such as the delivery of immune checkpoint inhibitors (anti-PD-1, anti-PD-L1, anti-CTLA4 [58]) or cytokines (IFN-a, IL-2 family, IL-12 and IL-10 [59], hold promise for activating anti-tumor immune responses and eliciting durable anti-cancer effects. Additionally, the long-term consequences of ctDNA integration into recipient cell genomes, including potential oncogenic effects or alterations in cellular function, need to be elucidated. KTS is in the process of delivering therapeutic small molecule drugs using “Zip-code” DNAs into cancer cells, with initial results showing very promising and encouraging outcomes. This concept can be applied to different types of cancers, as we are currently defining and discovering various cancer cell-specific Zip-codes and payloads. Due to the unique advantage of Zip-code DNAs, this targeted approach minimizes damage to healthy cells, enhances the efficacy of the treatment, and reduces side effects. Zip-code DNA delivery systems hold promise for more personalized and precise cancer therapies. Moving forward, rigorous preclinical studies are essential to evaluate the safety, efficacy, and therapeutic potential of zip code-based gene therapy strategies in relevant cancer models. Subsequently, well-designed preclinical trials are warranted to assess these novel approaches’ feasibility, tolerability, and clinical benefit in cancer patients. Long-term follow-up studies are crucial for monitoring treatment outcomes, assessing potential side effects, and optimizing therapeutic protocols for widespread clinical application.

## Beyond oncology

**Figure 2:**
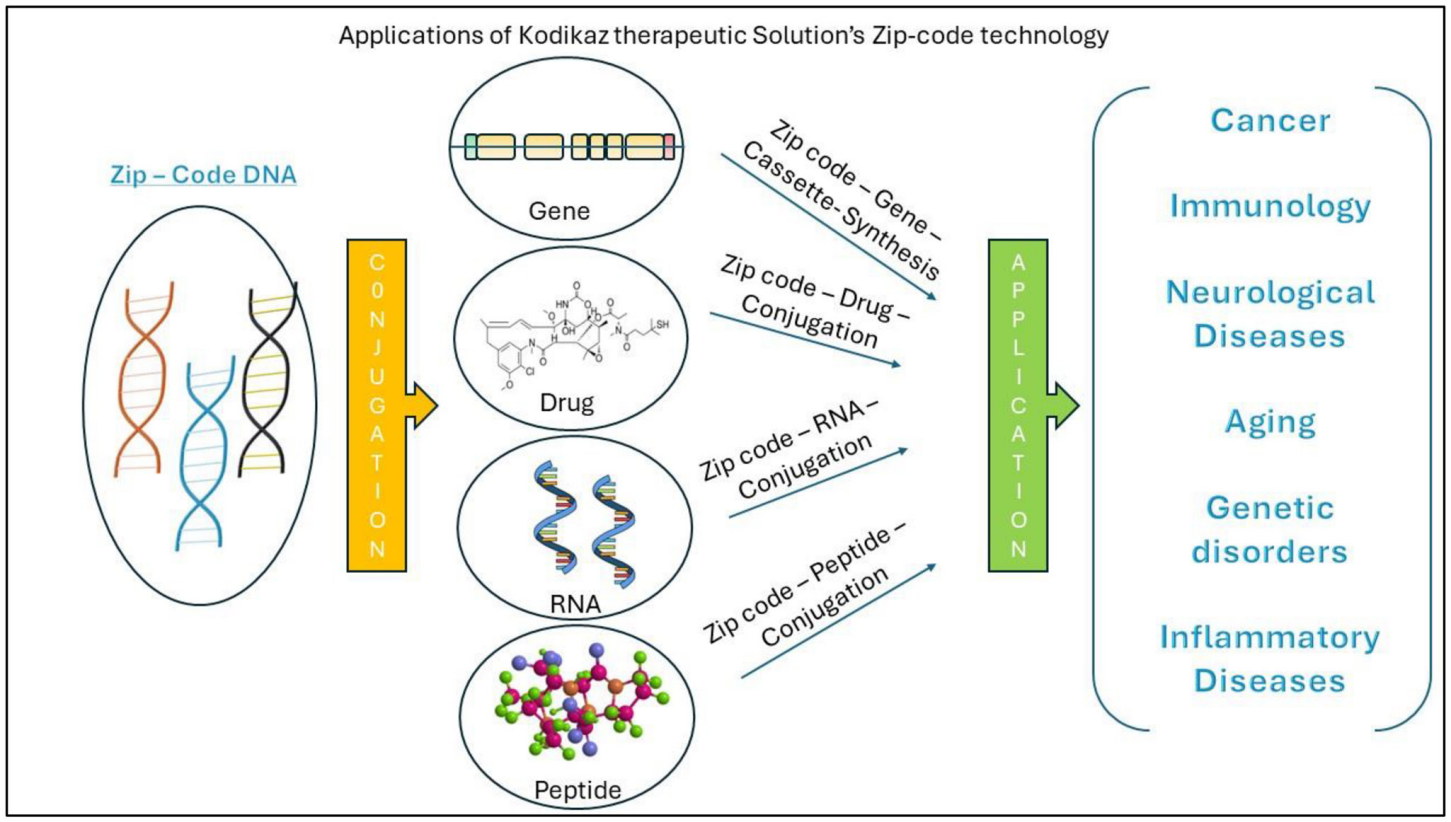
Diagrammatic representation of kodikaz therapeutic solutions’ zip-code technology application to various human diseases.

While much of the initial research on zip-code technology has focused on its applications in oncology, the potential applications extend far beyond cancer therapy. The disease that underlies cancer and the involvement of transposons are mimicked and have been implicated in other areas such as immunology, neuro and inflammatory diseases. Zip-code-based delivery systems could be adapted for targeted delivery of therapeutics in these disease contexts, as well as infectious diseases, neurodegenerative disorders, autoimmune conditions and genetic disorders (Figure 2). Furthermore, the development of zip-code technology for genetic engineering and gene editing holds promises for applications in regenerative medicine and others. As research in this field advances, interdisciplinary collaborations between oncologists, immunologists, geneticists, and engineers will be essential for translating zip-code technology into clinical practice across diverse medical specialties.

## CONCLUSIONS

This research work sheds new light on the functional significance of retrotransposon-derived DNA zip codes in myeloma cell biology and tumor microenvironment interactions. By elucidating the mechanisms underlying DNA internalization and its implications for cancer progression, the study provides a foundation for future research to exploit this process for therapeutic intervention in multiple myeloma, other malignancies and beyond. Investigating zip code DNAs for developing novel non-viral gene therapy strategies represents a promising frontier in cancer research. By harnessing the unique targeting capabilities of zip code motifs, researchers can overcome barriers to gene delivery and revolutionize cancer treatment. Additionally, the study represents a paradigm shift in our understanding of genetic exchange in human cancers, highlighting the pivotal role of ctDNA-mediated GT in tumor evolution and therapy. By unraveling the molecular mechanisms underlying this phenomenon and demonstrating its therapeutic potential, the research paves the way for future studies aimed at harnessing qualities of ctDNA as a tool for precision cancer therapy and advancing our knowledge of cancer biology. As exemplified by this research, Zip-code technology offers unprecedented opportunities for precise delivery of therapeutics to target cells. By harnessing the tissue-specific tropism of ctDNA and its associated retrotransposons, zip-code-based delivery systems hold promise for transforming cancer therapy, immunoengineering, and beyond. Collaboration between academia, industry, and regulatory agencies will be essential for translating zip-code technology from the bench to the bedside and realizing its full potential in improving patient outcomes and advancing human health.
